# A National System of Mortality Registration

**Published:** 1899-11

**Authors:** W. A. King

**Affiliations:** Chief Statistician; Department of the Interior, Census Office, Washington, D. C.


### A National System of Mortality Registration.

DEPARTMENT OF THE INTERIOR,
CENSUS OFFICE,
WASHINGTON, D. C., September 22, 1899.

*Editor Texas Medical Journal, Austin, Texas.*

DEAR SIR: Appreciating the important aid your influential journal can give the Census Office in its present effort to secure the adoption of a uniform certificate for the return of deaths and looking toward the establishment of a national system of mortality registration, the Director of the Census has instructed me to call your attention to the circular (7-207) enclosed, and to prepare for you the accompanying condensed statement of facts (7-234) contained in the former, which is intended for publication, if you feel warranted in publishing it.

The circular fully details the steps that have been taken in this direction by the Census Office and shows that nearly all of the registration officers, with whom correspondence has been had, have courteously and promptly given the assurance desired. They promise generally to amend or recast their local death certificates so as to embrace all the census requirements, and to put the improved form in use for the calendar year 1900, which is the period selected for the collection of mortality returns.

Coöperation has thus been practically secured, which is all that can be accomplished by the office in this direction, but this, in itself, does not insure the satisfactory return of the information required for the census statistics. Something further is necessary.

It is apparent that upon the medical fraternity of the United States, more than upon any other agency involved, depends the ultimate success of the project, as the prompt and correct return, upon the new death certificates, of the facts required by Congress for the census, is the chief essential. The Director believes that their active coöperation and interest in a reform that promises such results to the cause of science may be relied upon if the journals that so thoroughly disseminate medical knowledge bring it to the attention of their readers.

Trusting that your journal will do all that it can to enlist its clientele of physicians in promoting national uniformity, and directing your especial attention to the concluding paragraph of the circular, I have the honor to be,

Very respectfully,
W. A. KING, Chief Statistician.

